# Molecular and Cellular Effects of Therapies for Thyroid Eye Disease on Ocular Surface and Adnexal Homeostasis

**DOI:** 10.3390/cells15070622

**Published:** 2026-03-31

**Authors:** Monika Sarnat-Kucharczyk, Wojciech Luboń, Dorota Wyględowska-Promieńska, Adrian Smędowski

**Affiliations:** 1 Department of Ophthalmology, Faculty of Medical Sciences, Medical University of Silesia, 40-514 Katowice, Poland; wlubon@sum.edu.pl (W.L.); dwygledowska@sum.edu.pl (D.W.-P.); asmedowski@sum.edu.pl (A.S.); 2 Department of Ophthalmology, Professor K. Gibiński University Clinical Center, Medical University of Silesia, 40-514 Katowice, Poland; 3 Department of Pediatric Ophthalmology, Faculty of Medical Sciences, Medical University of Silesia, 40-514 Katowice, Poland; 4 GlaucoTech Co., Ltd., 40-282 Katowice, Poland

**Keywords:** thyroid eye disease, ocular surface, molecular mechanisms, cellular signaling, inflammation, cytokines, meibomian gland dysfunction, tear film, orbital radiotherapy, teprotumumab

## Abstract

**Highlights:**

**What are the main findings?**

- Thyroid eye disease (TED)-associated ocular surface dysfunction arises from combined immune activation, epithelial stress, glandular impairment, and exposure-related mechanical factors.
- Systemic therapies for TED reduce orbital inflammation but have heterogeneous and incompletely characterized effects on ocular surface and adnexal homeostasis.
- Targeted biologic therapies primarily modulate orbital inflammatory signaling, while direct evidence for improvement in ocular surface homeostasis remains limited.
- Corticosteroids provide rapid anti-inflammatory effects but may impair epithelial repair and tear film stability.
- Orbital radiotherapy may induce oxidative and glandular stress contributing to meibomian gland dysfunction and dry eye symptoms.

**What are the implications of the main findings?**

- Current therapies may have variable or limited effects on ocular surface health in TED.
- Integrated management combining disease-modifying therapy with ocular surface-directed care is important for optimizing long-term outcomes.

**Abstract:**

Thyroid eye disease (TED) is an autoimmune inflammatory disorder primarily affecting orbital tissues, but ocular surface and adnexal involvement represent a frequent and clinically significant component of disease burden. Beyond mechanical exposure resulting from eyelid retraction and proptosis, TED-associated ocular surface disease arises from complex interactions between immune activation, epithelial stress, glandular dysfunction, and altered neuro-epithelial signaling. Increasing use of systemic immunomodulatory therapies, biologics, and orbital radiotherapy has improved control of orbital inflammation; however, their molecular and cellular effects on ocular surface homeostasis remain incompletely defined. This review summarizes current evidence on the cellular and molecular mechanisms underlying ocular surface dysfunction in TED and examines how disease-modifying therapies influence epithelial integrity, tear film stability, meibomian and lacrimal gland function, and local immune signaling. Key pathways discussed include cytokine-mediated inflammation, thyroid-stimulating hormone receptor and insulin-like growth factor-1 receptor crosstalk, pro-fibrotic signaling, neuro-inflammatory mechanisms, and epithelial stress responses involving mitogen-activated protein kinase and nuclear factor kappa B pathways. We further highlight the challenge of disentangling therapy-induced molecular effects from persistent exposure-related mechanical stress. Understanding how TED therapies modulate ocular surface and adnexal homeostasis is essential for optimizing integrated management strategies that address both orbital inflammation and long-term ocular surface stability.

## 1. Introduction

Thyroid eye disease (TED) is an autoimmune inflammatory disorder involving the orbital and periocular tissues, most commonly associated with Graves’ disease [1]. The condition is characterized by eyelid retraction, periorbital edema, conjunctival hyperemia, and proptosis, and in advanced cases may lead to compressive optic neuropathy with consequent visual impairment. In addition to these hallmark orbital manifestations, a substantial proportion of patients develop ocular surface disease, presenting with symptoms of dryness, irritation, foreign body sensation, and visual fluctuation [2]. Ocular surface involvement, most commonly presenting as dry eye disease (DED), is among the most frequent manifestations of TED and often precedes the development of classic orbital signs, with reported prevalence rates reaching up to 65.2% [3,4]. Although the association between TED and DED is well documented, the underlying mechanisms linking these conditions remain incompletely understood. Structural changes characteristic of TED, including widening of the palpebral fissure and eyelid abnormalities, are thought to disrupt ocular surface homeostasis by increasing corneal exposure and altering blink dynamics [5]. These mechanical alterations contribute to tear film instability and promote ocular surface inflammation, thereby facilitating the development of DED in patients with TED [6,7].

Beyond orbital manifestations, TED is a complex immune-mediated condition in which inflammatory signaling extends beyond the orbit to the ocular surface [8]. Elevated tear levels of interleukins (IL): IL-1β, IL-6, IL-8, IL-10, IL-17A, and tumor necrosis factor alpha (TNF-α) in active TED indicate a complex inflammatory milieu at the ocular surface. IL-1β may contribute to T-cell differentiation, whereas IL-8 promotes neutrophil chemotaxis, highlighting inflammation as a key contributor to TED-associated DED [8,9,10]. The ocular surface is an anatomically distinct but immunologically active tissue composed of epithelial, glandular, and immune cells that participate in inflammatory and homeostatic processes [2]. These features may render it particularly susceptible to systemic immunomodulatory therapies.

Importantly, long-term immunosuppression or orbital radiotherapy commonly used in the management of TED may further compromise an already vulnerable ocular surface [11]. In the setting of pre-existing ocular surface disease, these treatments can exacerbate tear film instability, epithelial dysfunction, and inflammatory damage, thereby amplifying dry eye symptoms and patient discomfort [12]. Despite the increasing use of immunomodulatory therapies in TED, their impact on ocular surface homeostasis remains insufficiently defined. In particular, the effects of these treatments on epithelial integrity, glandular function, and local immune signaling are not well characterized.

Despite the growing recognition of ocular surface involvement in TED, the specific molecular and cellular mechanisms linking systemic TED therapies with ocular surface and adnexal homeostasis remain insufficiently characterized. In particular, it remains unclear to what extent disease-modifying treatments modulate epithelial barrier integrity, tear film stability, and glandular function at the ocular surface. Therefore, the aim of this review is to summarize current evidence on the molecular and cellular pathways underlying ocular surface dysfunction in TED and to examine how commonly used systemic therapies influence ocular surface and adnexal homeostasis.

## 2. Ocular Surface Pathophysiology in Thyroid Eye Disease

### 2.1. Key Cell Types and Their Interactions at the Ocular Surface

Ocular surface disease (OSD) in TED arises from a multifactorial disruption of the cellular and molecular homeostasis of the tear film, corneal epithelium, conjunctiva, meibomian gland dysfunction (MGD) and accessory lacrimal glands [5]. This pathological process reflects a coordinated dysfunction of epithelial, secretory, and immune compartments, ultimately leading to tear film instability and persistent ocular surface inflammation.

#### 2.1.1. Corneal and Conjunctival Epithelial Cells

Corneal and conjunctival epithelial cells serve as both physical barriers and active immunoregulatory elements of the ocular surface. In TED, increased ocular exposure due to eyelid retraction and proptosis induces epithelial stress, leading to the upregulation of inflammatory mediators such as IL-1β, IL-6, IL-8, TNF-α, and matrix metalloproteinases. These factors promote epithelial dysfunction, impaired wound healing, and increased corneal permeability.

Corneal confocal microscopy studies have revealed increased keratocyte activation and decreased corneal nerve fiber density in active TED [13,14]. These changes may disrupt corneal neural feedback mechanisms and further compromise ocular surface homeostasis.

#### 2.1.2. Conjunctival Goblet Cells

Conjunctival goblet cells, which are essential for mucin secretion and tear film stability, are reduced in density and function in TED-associated OSD. Loss of goblet cells results in decreased mucin production, contributing to tear film instability and increased friction at the ocular surface. Concurrently, lacrimal gland acinar cells may exhibit functional impairment secondary to inflammatory signaling, further exacerbating aqueous tear deficiency. Conjunctival impression cytology, tear proteomics studies and confocal microscopy [15,16,17] demonstrate that ocular surface damage is more pronounced in active TED than in inactive disease, reflecting the combined effects of orbital inflammation and mechanical stress.

#### 2.1.3. Meibomian Glands

MGD, a major contributor to evaporative DED, may share overlapping pathogenic mechanisms with TED-associated ocular surface disease. At the cellular level, normal meibum secretion depends on coordinated differentiation and holocrine secretion of meibocytes, processes that are facilitated by effective blinking–induced shear forces [18]. In TED, proptosis and eyelid retraction impair blink completeness, leading not only to mechanical obstruction of gland orifices but also to altered meibocyte turnover and lipid secretion [6,7]. These changes promote obstructive MGD, destabilization of the tear film lipid layer, and sustained ocular surface stress, thereby contributing to dry eye development in TED [7].

Barman, Kakil, and Arslan reported substantial ocular surface dysfunction in TED, characterized by reduced tear break-up time (TBUT), increased symptom burden as measured by the OSDI, and corneal staining, with significantly worse and more persistent dry eye manifestations in smokers. Over follow-up, treatment was associated with significant improvements in TBUT, OSDI, and Oxford staining scores, while selenium supplementation showed potential benefit and smoking cessation was highlighted as a key modifiable factor for improving outcomes [1].

#### 2.1.4. Lacrimal Glands

The lacrimal gland has been implicated as a direct target in the pathogenesis of DED in thyroid patients with glandular enlargement observed in hyperthyroid TED [19]. Expression of thyroid-stimulating hormone receptor (TSHR) in lacrimal gland acinar epithelial cells suggests susceptibility to autoimmune attack, leading to dacryoadenitis, which is widely prevalent and commonly underdiagnosed. Ultimately, fibrotic remodeling and lacrimal gland dysfunction occur [20].

Immune cell involvement is a critical component of ocular surface pathology in TED. CD4^+^ T helper cells, particularly Th1 and Th17 subsets, together with macrophages, and mast cells infiltrate the conjunctival and peri-lacrimal tissues, where they release pro-inflammatory cytokines and chemokines that perpetuate epithelial damage and goblet cell loss. The reciprocal interactions between epithelial cells and immune cells establish a chronic inflammatory microenvironment, reinforcing tear film dysfunction and sustaining ocular surface inflammation.

### 2.2. Molecular Signaling Pathways

Ocular surface disease in TED results from immune, receptor-mediated, and mechanical processes that impair tear secretion and compromise ocular surface integrity. Chronic inflammation, maintained by immune cell activation and epithelial stress, is a key driver of disease progression [21,22].

#### 2.2.1. Cytokine-Mediated Inflammatory Signaling

Elevated tear levels of IL-1β, IL-6, IL-8, and TNF-α are a consistent feature of active TED, reflecting sustained ocular surface inflammation [23]. These cytokines engage nuclear factor kappa B (NF-κB) and mitogen-activated protein kinase (MAPK)-dependent signaling pathways in epithelial cells through receptor-mediated activation of intracellular kinase cascades. Activation of MAPK components such as ERK, c-Jun N-terminal kinase (JNK), and p38 promotes barrier disruption, goblet cell depletion, and upregulation of matrix metalloproteinases [24,25]. Persistent cytokine signaling maintains a pro-inflammatory microenvironment that destabilizes the tear film and impairs epithelial repair processes.

#### 2.2.2. TSHR–IGF-1R Signaling Interactions

TSHR and insulin-like growth factor-1 receptor (IGF-1R) play a central role in TED pathophysiology and are increasingly implicated in ocular surface involvement.

The functional crosstalk between TSHR and IGF-1R is most prominent in CD34^+^ Graves’ disease orbital fibroblasts, where high receptor expression drives inflammatory signaling, whereas CD34^−^ fibroblasts exhibit weaker receptor coupling and predominantly contribute to tissue remodeling [26,27,28].

The axon guidance molecule Slit2 and its cognate receptor Roundabout homolog 1 (ROBO1), classically involved in neuronal development, have emerged as important regulators of immune and inflammatory responses. In TED, orbit-infiltrating CD34^+^ fibrocytes express ROBO1, while Slit2 is produced within the orbital microenvironment. Engagement of ROBO1 by Slit2 modulates the inflammatory phenotype of these fibrocytes by attenuating pro-inflammatory signaling pathways [26].

Slit2–ROBO1 signaling modulates the inflammatory phenotype of CD34^+^ orbit-infiltrating fibrocytes in TED by suppressing NF-κB-dependent cytokine production [26]. This pathway functions as an intrinsic regulator of inflammation and intersects with TSHR–IGF-1R signaling, limiting excessive receptor-driven immune activation [27]. Impaired Slit2–ROBO1 signaling may contribute to the exaggerated inflammation observed in active TED by permitting unrestrained TSHR–IGF-1R-mediated signaling in CD34^+^ orbital fibroblasts. Accordingly, the Slit2–ROBO1 axis represents a potential immunomodulatory pathway relevant to disease activity and therapeutic intervention [26,27].

#### 2.2.3. Pro-Fibrotic Pathways and Tissue Remodeling

Chronic inflammation in TED activates pro-fibrotic signaling pathways, most notably those mediated by transforming growth factor-β (TGF-β) [29,30]. TGF-β signaling promotes extracellular matrix deposition, myofibroblast differentiation, and fibrotic remodeling within periocular tissues and the lacrimal gland [31,32]. Such remodeling results in irreversible structural alterations, leading to persistent lacrimal gland dysfunction and long-term aqueous tear deficiency.

#### 2.2.4. Neuro-Inflammatory and Mechanical Stress Signaling

Alterations in corneal innervation observed in active TED disrupt neuro-epithelial feedback mechanisms involved in the regulation of tear secretion and blink reflexes [33,34]. Reduced corneal nerve density and impaired sensory input result in decreased release of neuropeptides, including substance P and calcitonin gene-related peptide (CGRP), and vasoactive intestinal peptide (VIP), as well as diminished neurotrophic support mediated by nerve growth factor (NGF), thereby enhancing epithelial stress and inflammatory signaling [35,36,37], In parallel, increased ocular exposure and incomplete blinking activate epithelial mechanotransduction pathways, including integrin–focal adhesion kinase (FAK) signaling and MAPK/NF-κB cascades, further promoting pro-inflammatory cytokine production and epithelial degeneration at the ocular surface [38,39].

In vivo confocal microscopy has increasingly been applied to the evaluation of ocular surface disease in TED and has provided evidence supporting a pro-inflammatory disease model. Villani et al. demonstrated that patients with TED exhibit reduced corneal epithelial cell density, increased stromal keratocyte activation, and decreased corneal nerve density compared with healthy controls [14,40].

## 3. Biologic Therapies

### 3.1. IGF-1R Inhibition (Teprotumumab)

#### 3.1.1. Molecular Mechanism of Receptor-Level Interference

Teprotumumab is a monoclonal antibody that was originally developed for oncologic indications but has since been repurposed for the treatment of TED. The therapeutic effect of teprotumumab is mediated through partial antagonism of the IGF-1R, which is known to co-localize with the TSHR on orbital fibroblasts. By binding to the α-subunit of IGF-1R, teprotumumab inhibits receptor signaling via three complementary mechanisms: (i) promotion of IGF-1R internalization and subsequent degradation, (ii) direct blockade of IGF-1R activation, and (iii) disruption of IGF-1R–TSHR complex formation [2]. As a consequence, teprotumumab suppresses IGF-1R-mediated, TSH-induced synthesis of pro-inflammatory cytokines, including IL-6 and IL-8, thereby exerting a net anti-inflammatory effect [41].

#### 3.1.2. Downstream Inflammatory Modulation

Teprotumumab-mediated IGF-1R blockade has been shown to attenuate orbital fibroblast activation and reduce hyaluronan synthesis, thereby limiting tissue edema and extracellular matrix expansion [25]. In addition, inhibition of IGF-1R signaling appears to interfere with adipogenic differentiation of orbital fibroblasts, contributing to decreased orbital tissue remodeling and inflammatory amplification [28].

#### 3.1.3. Potential Implications for Ocular Surface Homeostasis

Although systemic IGF-1R inhibition may indirectly modulate ocular surface inflammation, direct evidence of improved tear film stability or epithelial homeostasis in TED remains limited. Thus, potential ocular surface benefits of teprotumumab are currently extrapolated from systemic immunologic effects rather than surface-specific clinical endpoints [2].

### 3.2. IL-6 Receptor Blockade (Tocilizumab)

#### 3.2.1. IL-6-Dependent Signaling Pathways

IL-6 signaling contributes to inflammatory activation and tissue remodeling in TED through downstream pathways such as JAK/STAT, MAPK, and NF-κB. IL-6 receptor antagonists, including tocilizumab, suppress these pathways and have demonstrated efficacy in active, steroid-resistant disease, highlighting IL-6 as a key therapeutic target [42].

#### 3.2.2. Ocular Surface Implications and Current Evidence Gap

IL-6 signaling has been implicated in fibroblast activation and pro-fibrotic remodeling through STAT3-dependent pathways and crosstalk with TGF-β. While IL-6 receptor blockade with tocilizumab effectively reduces inflammatory activity in steroid-resistant TED, direct evidence demonstrating attenuation of orbital fibrosis or ocular surface remodeling remains limited [25,29].

### 3.3. B-Cell Depletion (Rituximab)

#### 3.3.1. Immunologic Mechanism

Rituximab, a monoclonal antibody targeting CD20 on B cells, depletes pathogenic B-lymphocytes and exerts immunomodulatory effects in TED. While its systemic anti-inflammatory action may theoretically benefit chronic ocular surface inflammation, direct evidence on rituximab’s effects on tear film stability, epithelial homeostasis, or meibomian gland function in TED is currently lacking, highlighting an important gap in mechanistic understanding and clinical evaluation [2].

In a prospective open-label trial of 12 patients with active TED (CAS ≥ 4), rituximab (1000 mg administered twice, 2 weeks apart) induced rapid peripheral B-cell depletion and was associated with sustained reductions in CAS over 12 months. The treatment was generally well tolerated, although efficacy interpretation was limited by the variable natural course of TED [43].

#### 3.3.2. Clinical Data and Ocular Surface Considerations

Beyond antibody depletion, B-cell targeting with rituximab may also modulate antigen presentation and downstream T-cell activation, thereby contributing to broader immunoregulatory effects in TED; however, direct implications for ocular surface homeostasis have not been systematically evaluated [2].

Theoretically, B-cell depletion may influence tear cytokine profiles and conjunctival immune cell populations through broader immunoregulatory effects; however, direct evidence in TED remains lacking [2].

**Figure 1:**
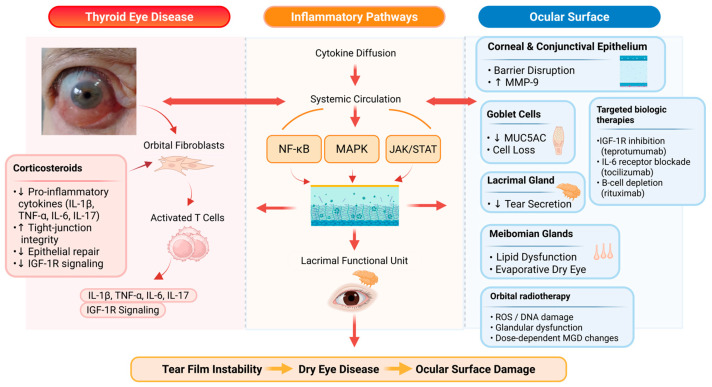
Molecular pathways of ocular surface dysfunction in Thyroid Eye Disease. Orbital immune activation in TED involves orbital fibroblasts and infiltrating T cells that produce pro-inflammatory cytokines, including IL-1β, TNF-α, IL-6, and IL-17, and activate IGF-1R-dependent signaling. These mediators stimulate intracellular inflammatory pathways such as NF-κB, MAPK, and JAK/STAT signaling. The resulting inflammatory cascade disrupts ocular surface homeostasis, leading to epithelial barrier damage and increased MMP-9 expression, loss of conjunctival goblet cells with reduced MUC5AC production, impaired lacrimal gland aqueous secretion, and meibomian gland lipid secretion. The figure also illustrates the mechanisms of action of systemic therapies used in TED, including corticosteroids, targeted biologic therapies (teprotumumab, tocilizumab, rituximab), and orbital radiotherapy. The combined effects of inflammation, glandular dysfunction, and epithelial damage contribute to tear film instability, DED, and progressive ocular surface injury. Some molecular pathways illustrated are supported by direct evidence from ocular surface studies, whereas others are extrapolated from orbital fibroblast or orbital tissue data due to limited surface-specific mechanistic evidence in TED. Black upward arrows (↑) indicate an increase, black downward arrows (↓) indicate a decrease, red arrows indicate the direction of signaling pathways and interactions, orange arrows indicate systemic circulation and bidirectional interactions, and light orange arrows indicate disease progression. Created in BioRender. Monika Sarnat-Kucharczyk. (2026) https://BioRender.com/hjfebsd.

Figure 1 summarizes the molecular pathways linking TED-associated inflammation with ocular surface and adnexal dysfunction and highlights the differential mechanisms of action of systemic corticosteroids, targeted biologic therapies, and orbital radiotherapy.

## 4. Systemic Corticosteroids

### 4.1. Mechanisms of Action

Systemic corticosteroids remain a cornerstone therapy for active, moderate-to-severe TED, providing rapid anti-inflammatory and immunosuppressive effects that improve orbital congestion and soft-tissue inflammation [42]. By attenuating cytokine-mediated vascular permeability, they reduce tissue edema and alleviate acute inflammatory signs. Nevertheless, corticosteroid therapy is associated with a broad spectrum of adverse effects, including clinically relevant disturbances of ocular surface and adnexal homeostasis [44].

### 4.2. Suppression of Innate Immune Defense

Systemic glucocorticoids activate the glucocorticoid receptor (GR; NR3C1), which translocates to the nucleus and suppresses inflammatory gene expression through transrepression of NF-κB- and AP-1-dependent signaling pathways. Although this mechanism effectively reduces ocular surface inflammation, it may concurrently impair epithelial innate immune functions by downregulating antimicrobial peptides and other barrier-associated defense mediators, such as β-defensins, cathelicidin (LL-37), and lactoferrin [45]. In addition, glucocorticoids can attenuate chemokine-driven leukocyte recruitment, thereby weakening local immune surveillance and increasing susceptibility to secondary infections caused by bacteria, viruses, and fungi [44].

Glucocorticoid signaling in human corneal epithelial cells markedly alters gene expression and cellular behavior, and dexamethasone was shown to delay epithelial wound closure primarily by reducing cell migration and inducing cytoskeletal remodeling at the wound edge. Interestingly, corticosteroid exposure may concurrently improve epithelial barrier function by enhancing tight-junction organization, indicating a trade-off between impaired repair and strengthened barrier integrity [46].

### 4.3. Impaired Epithelial Proliferation and Wound Healing

Glucocorticoids can suppress epithelial cell-cycle progression and attenuate growth factor-dependent repair responses by modulating epidermal growth factor receptor (EGFR)–MAPK/extracellular signal-regulated kinase (ERK) signalling and shifting transcriptional programs toward an anti-proliferative phenotype. Consequently, corneal epithelial wound closure may be delayed, particularly in patients with exposure keratopathy or pre-existing epithelial compromise [47].

It should be noted that several mechanistic insights regarding corticosteroid effects on epithelial repair and junctional integrity are derived from general corneal epithelial models rather than TED-specific studies and therefore represent extrapolated mechanisms that may contribute to ocular surface alterations in TED.

However, direct clinical evidence in TED patients demonstrating increased epithelial defects or infectious keratitis during systemic glucocorticoid therapy remains limited, and most available data relate to tear film alterations and ocular surface inflammation rather than clinically documented epithelial complications.

### 4.4. Barrier Dysfunction and Altered Epithelial Differentiation

By modulating GR-dependent transcription, corticosteroids may alter the expression of epithelial junctional proteins including zonula occludens-1 (ZO-1), occludin, and claudins, as well as differentiation markers. These changes may affect tight-junction organization and epithelial homeostasis. Under vulnerable ocular surface conditions (e.g., exposure-related stress), these changes could contribute to punctate epithelial erosions and increased epithelial fragility [46].

### 4.5. Reduced Goblet Cell Function and Mucin Availability (Tear Film Instability)

Systemic corticosteroids may reduce conjunctival immune activation. However, they may also affect goblet cell function and mucin-related homeostasis, potentially compromising MUC5AC-dependent tear film stability, which is central to tear film homeostasis [48,49]. Reduced goblet cell density and tear MUC5AC levels have been demonstrated in patients with TED, supporting the relevance of mucin deficiency in TED-associated ocular surface disease [48]. These findings are consistent with broader evidence highlighting the role of mucin barrier disruption in ocular surface disorders [49]. Clinically, this could contribute to tear film instability with increased tear film breakup and dry eye symptoms, particularly when TED-associated lid retraction and incomplete blinking persist [50].

### 4.6. Altered Inflammatory Resolution vs. Chronic Surface Stress

Although corticosteroids can acutely reduce pro-inflammatory cytokines such as IL-1β, IL-6, and TNF-α, the ocular surface in TED remains exposed to persistent mechanical and evaporative stress. This imbalance between dampened immune surveillance and ongoing epithelial stress may contribute to sustained epithelial injury despite reduced clinical signs of inflammation [51].

### 4.7. Effects on Adnexal Tissues: Meibomian Gland Dysfunction (MGD) and Lipid Layer Changes

Systemic corticosteroids may alter the lid margin microenvironment via GR-dependent effects on lipid metabolism and inflammatory signaling in the meibomian glands [52]. In TED patients with pre-existing MGD, this may destabilize the tear film lipid layer and aggravate evaporative dry eye and ocular surface hyperosmolarity [6].

Table 1 summarizes the differential molecular and cellular effects of systemic corticosteroids and biologic therapies on ocular surface and adnexal homeostasis in TED, highlighting distinct levels of intervention, signaling targets, and downstream consequences for epithelial integrity, tear film stability, and glandular function

## 5. Orbital Radiotherapy

### 5.1. Radiation-Induced Cellular Stress and DNA Damage Responses

Orbital radiotherapy may affect ocular surface homeostasis through radiation-induced oxidative stress and DNA damage, triggering cellular stress responses that promote epithelial and glandular dysfunction, including impaired proliferation and increased cell death [53].

Beyond its immunomodulatory effects on orbital inflammation, ionizing radiation induces a cascade of molecular stress responses in ocular surface and adnexal tissues. Radiation exposure promotes reactive oxygen species (ROS) generation and DNA damage, activating p53-dependent cell cycle arrest, mitochondrial dysfunction, and cellular senescence programs [54]. These processes disrupt epithelial proliferation and differentiation while simultaneously triggering NF-κB–mediated inflammatory signaling, thereby perpetuating a low-grade pro-inflammatory microenvironment despite clinical improvement in orbital activity [55,56,57].

Radiation-induced DNA damage activates ATM/ATR-dependent checkpoint signaling, amplifying oxidative stress through mitochondrial dysfunction and reinforcing senescence-associated inflammatory programs [58,59].

It should be noted that most of the molecular pathways described above, including radiation-induced DNA damage responses, mitochondrial dysfunction, and cellular senescence signaling, are primarily derived from broader radiation biology literature and have not been directly demonstrated in ocular surface tissues in TED.

### 5.2. Epithelial Injury and Impaired Regenerative Capacity

In TED, these effects may be clinically relevant given the baseline exposure-related stress and frequent co-existing tear film instability. Disruption of epithelial proliferation and differentiation contributes to impaired regenerative capacity of the ocular surface epithelium.

Radiation exposure reduces epithelial proliferative capacity and may deplete progenitor cell pools, contributing to delayed surface renewal and persistent epithelial fragility [60,61].

### 5.3. Glandular Dysfunction: Lacrimal Acinar Injury and Meibomian Gland Alterations

At the tissue level, these cellular stress responses may impair lacrimal acinar secretion and meibomian lipid synthesis and differentiation, leading to reduced aqueous and lipid tear components, tear film destabilization, and hyperosmolar epithelial stress [62]. In glandular tissues, radiation-induced oxidative stress impairs acinar cell secretory capacity and alters lipid metabolic pathways in meibomian glands, contributing to reduced aqueous output and destabilization of the tear film lipid layer [63,64].

In an observational case–control study, Chen et al. demonstrated that unilateral orbital radiotherapy (median dose 45 Gy) was associated with significant bilateral meibomian gland damage, reflected by increased gland loss, reduced lipid layer thickness, shortened TBUT, and higher OSDI scores compared with healthy controls [63]. Notably, meibomian gland loss and corneal staining correlated with radiation dose, suggesting a dose-dependent impact of orbital radiotherapy on ocular surface stability [63].

### 5.4. Neuro-Epithelial Disruption and Altered Tear Reflexes

Importantly, radiation injury may also affect corneal sensory nerves and peri-glandular stromal cells, further compromising neuro-epithelial feedback mechanisms essential for tear secretion and epithelial maintenance [65].

### 5.5. Clinical Correlates: Tear Film Instability and Persistent Dry Eye Symptoms

In a prospective observational study of 16 patients (32 eyes) with active TED, steroid pulse therapy combined with orbital radiotherapy significantly improved clinical activity score (CAS) and reduced upper eyelid retraction. Nevertheless, while MGD parameters showed modest improvement, most objective and subjective dry eye measures remained largely unchanged at 6 months, suggesting limited short-term impact of this regimen on ocular surface status [66].

Importantly, radiation-related ocular surface changes may evolve gradually over months and correlate with cumulative dose, often progressing subclinically before becoming symptomatic [67]. However, longitudinal imaging or biomarker studies specifically evaluating subclinical progression of ocular surface changes in TED following orbital radiotherapy remain limited.

Together, these molecular and cellular alterations provide a mechanistic framework explaining why orbital radiotherapy, while effective in reducing orbital inflammation, may exert persistent adverse effects on ocular surface homeostasis, particularly in patients with pre-existing exposure-related stress and MGD [68].

To contextualize the heterogeneity of available data, Table 2 summarizes the strength and type of evidence supporting therapy-related effects on ocular surface and adnexal homeostasis in TED, distinguishing between direct clinical surface endpoints and indirect or mechanistic data.

For biologic therapies, evidence is largely derived from orbital outcomes and mechanistic reviews, as direct ocular surface endpoints are currently lacking.

To provide a consolidated overview of currently available clinical evidence, Table 2 summarizes studies reporting ocular surface outcomes in patients undergoing different TED therapies.

## 6. Clinical Implications and Management Strategies

Ocular surface-directed management in patients with TED should be aligned with established DED frameworks, including the TFOS DEWS III recommendations [5], emphasizing tear film stabilization, management of MGD, and protection of epithelial barrier integrity.

**Figure 2:**
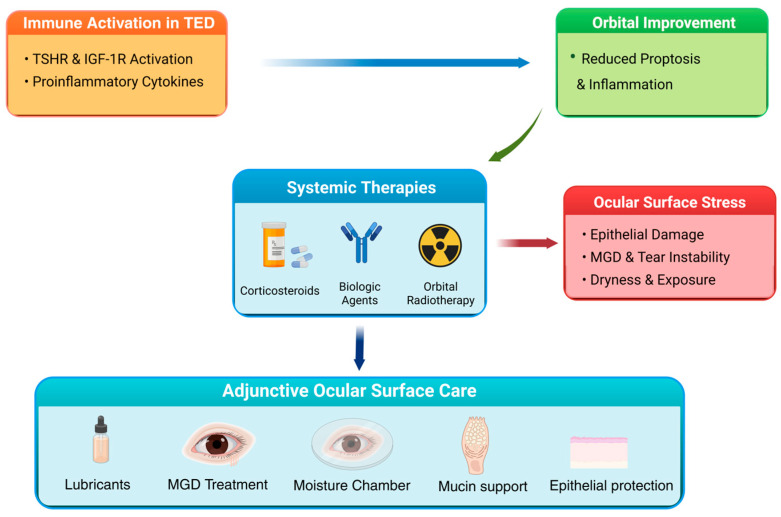
Clinical Treatment Algorithm for Thyroid Eye Disease illustrating the relationship between immune activation, systemic therapies, and ocular surface management. Immune activation in TED is driven by TSHR and IGF-1R signaling and the production of pro-inflammatory cytokines, leading to orbital inflammation and proptosis. Systemic therapies, including corticosteroids, biologic agents, and orbital radiotherapy, aim to reduce orbital inflammation and improve orbital manifestations. However, despite orbital improvement, patients may continue to experience ocular surface stress due to epithelial damage, meibomian gland dysfunction, tear film instability, and exposure-related dryness. Adjunctive ocular surface therapies, including lubricants, treatments targeting meibomian gland dysfunction, moisture chamber strategies, mucin-supportive therapies, and epithelial protective measures, are therefore essential components of comprehensive TED management. Blue arrows indicate the direction of processes (e.g., immune activation leading to orbital improvement), green arrows indicate process effects, red arrows indicate effects on ocular surface stress, and dark blue downward arrows indicate progression toward adjunctive ocular surface therapy. Created in BioRender. Monika Sarnat-Kucharczyk. (2026) https://BioRender.com/hjfebsd.

Based on current evidence, we propose an integrated therapeutic framework (Figure 2) highlighting how systemic disease-modifying therapies improve orbital inflammation while persistent exposure-related stress necessitates parallel ocular surface–directed management.

Despite effective control of orbital inflammation, many patients with TED continue to exhibit ocular surface instability driven by persistent exposure-related mechanical stress, tear film hyperosmolarity, and co-existing MGD [21,22]. Clinical management should therefore integrate systemic disease-modifying therapy with proactive ocular surface protection, including preservative-free lubrication and strategies to reduce exposure in patients with lid retraction or lagophthalmos [1,69]. MGD should be actively addressed to improve tear film lipid layer stability and mitigate evaporative stress [1]. Importantly, therapy-specific monitoring remains relevant, as systemic corticosteroids may delay epithelial repair and reduce host defense, while orbital radiotherapy may variably affect ocular surface structures in susceptible eyes [47].

Nevertheless, it remains challenging to disentangle therapy-induced molecular effects from exposure-driven mechanical stress in TED. While immunomodulatory therapies effectively suppress inflammatory signaling, persistent eyelid retraction and tear film hyperosmolarity continue to activate epithelial stress pathways, including MAPK and NF-κB signaling, thereby limiting full restoration of ocular surface homeostasis [70,71].

## 7. Discussion

The present review integrates emerging evidence linking immune-mediated orbital inflammation in TED with secondary alterations of the ocular surface and adnexal tissues. While structural factors such as eyelid retraction and increased ocular exposure have traditionally been considered the primary drivers of dry eye symptoms in TED, growing evidence indicates that inflammatory signaling also plays a significant role in ocular surface dysfunction. Elevated tear cytokines, epithelial barrier disruption, and alterations in corneal innervation collectively contribute to tear film instability and chronic ocular surface inflammation [9,10,21].

Importantly, the relationship between systemic TED therapies and ocular surface homeostasis appears to be complex. Although treatments such as corticosteroids, biologic agents, and orbital radiotherapy are effective in reducing orbital inflammation, their direct and indirect effects on ocular surface tissues remain incompletely characterized [2,42]. Some therapeutic interventions may improve inflammatory activity while simultaneously influencing epithelial repair, glandular function, or local immune regulation. As a result, patients may continue to experience ocular surface symptoms despite improvement in orbital disease activity.

Another important consideration is the limited availability of studies directly evaluating ocular surface outcomes in TED-specific treatment cohorts. Many mechanistic insights are derived from general ocular surface or corneal epithelial models and therefore represent extrapolated mechanisms rather than disease-specific evidence [24,46]. This highlights the need for more integrated clinical and translational studies evaluating both orbital and ocular surface endpoints in patients undergoing TED therapy.

Taken together, these findings emphasize that effective management of TED should consider not only orbital inflammation but also the complex interactions between immune signaling, epithelial integrity, glandular function, and tear film stability [21,22].

## 8. Future Directions

Future research should aim to disentangle therapy-specific molecular effects from exposure-driven ocular surface stress in TED, as clinical improvement in orbital inflammation does not necessarily translate into restoration of epithelial and tear film homeostasis. Mechanistic studies integrating single-cell or spatial transcriptomics with tear proteomics/lipidomics may clarify how systemic immunomodulation, orbital radiotherapy, and targeted biologics reshape conjunctival, corneal, and eyelid margin microenvironments. Particular attention should be directed toward key ocular surface cell populations involved in TED-associated dysfunction, including conjunctival goblet cells, corneal epithelial cells, meibomian gland acinar cells, lacrimal gland epithelial cells, and corneal sensory nerve-associated cellular niches. In addition, prospective longitudinal cohorts with standardized ocular surface endpoints are needed to define predictors of persistent dry eye, MGD progression, and epithelial fragility during and after TED treatment. Finally, translational strategies that combine disease-modifying therapy with barrier-supportive and lipid-stabilizing interventions should be evaluated to optimize both inflammatory control and long-term ocular surface resilience.

Beyond host-derived pathways, therapy-induced changes in ocular surface immunity and tear film composition may also reshape the ocular surface microbiome, with downstream effects on epithelial barrier function and inflammatory tone.

## 9. Conclusions

TED therapies reduce orbital inflammation, but ocular surface and adnexal homeostasis often remain impaired due to persistent exposure-related stress, tear film instability, and MGD. Integrating systemic treatment with proactive ocular surface management is therefore essential to optimize long-term outcomes.

## Figures and Tables

**Table 1:** Differential molecular and cellular effects of systemic corticosteroids and biologic therapies on ocular surface and adnexal homeostasis in TED.

Aspect	Systemic Corticosteroids	IGF-1R Inhibition (e.g., Teprotumumab)	IL-6 Pathway Blockade (e.g., Tocilizumab)	Rituximab
Level of intervention	Broad transcriptional regulation [44]	Receptor-level signaling	Cytokine receptor signaling	B-cell depletion
Primary target	GR/NR3C1	IGF-1R–TSHR signaling complex	IL-6 receptor	CD20^+^ B cells
Cytokine modulation	Broad suppression of pro-inflammatory cytokine expression via NF-κB and AP-1 transrepression [10,44]	Downstream suppression	Direct IL-6 pathway inhibition	Indirect
Epithelial repair	Enhanced tight-junction organization but delayed epithelial migration and wound healing [46]	Neutral/indirect	Neutral/indirect	Not directly studied
Goblet cell effects	Potential reduction in MUC5AC-related tear film stability [48,49]	No direct evidence	No direct evidence	Not directly studied
Meibomian gland effects	Possible destabilization of lipid metabolism and exacerbation of evaporative dry eye in predisposed patients [6,52]	Limited data	Limited data	Not directly studied
Ocular surface specificity	Rapid anti-inflammatory effect but risk of impaired epithelial repair and tear film instability [42]	Moderate (indirect)	Moderate (indirect)	Very limited surface data

**Table 2:** Clinical studies reporting ocular surface outcomes in patients undergoing TED therapies.

Therapy	Study	Study Design/Population	Ocular Surface Outcomes Assessed	Main Findings	Limitations
Systemic glucocorticoids	Xu et al. [10]	Clinical study in active TED treated with high-dose intravenous glucocorticoids	Tear inflammatory cytokines, ocular surface parameters	Glucocorticoid therapy was associated with reduced tear inflammatory cytokines and improvement in selected ocular surface findings	Limited direct assessment of epithelial complications; short-term ocular surface endpoints
Systemic glucocorticoids	Acar et al. [33]	Small open-label interventional case series in patients with active TED	Ocular surface findings, in vivo confocal microscopy	Improvement in ocular surface inflammatory findings and corneal nerve-related parameters after treatment	Limited sample size; no direct assessment of infectious complications
Biologic therapy (rituximab)	Silkiss et al. [43]	Prospective open-label study in active TED	No dedicated ocular surface endpoints	Reduction in CAS following rituximab therapy.	Ocular surface outcomes not systematically evaluated
Orbital radiotherapy	Chen et al. [64]	Observational case–control study	Meibomian gland loss, lipid layer thickness, TBUT, OSDI, corneal staining	Orbital radiotherapy was associated with meibomian gland damage, reduced lipid layer thickness, shortened TBUT, and higher OSDI scores.	Observational design; limited mechanistic correlation
Steroid pulse + orbital radiotherapy	Takahashi et al. [66]	Prospective observational study in active TED	Dry eye parameters, MGD measures	CAS improved, whereas most subjective and objective dry eye parameters showed little or no significant change at 6 months.	Small cohort; combined-treatment design limits attribution of effects
General TED management/mixed treatment context	Barman Kakil and Arslan [1]	Clinical observational study	TBUT, OSDI, corneal staining	Treatment was associated with improvement in TBUT, OSDI, and corneal staining; smoking worsened and prolonged dry eye manifestations	Not focused on one defined systemic therapy mechanism

## Data Availability

No new data were created or analyzed in this study. Data sharing is not applicable to this article.

## Abbreviations

The following abbreviations are used in this manuscript:

**Abbreviation**	**Full term**
CAS	Clinical Activity Score
CGRP	Calcitonin Gene-Related Peptide
DED	Dry Eye Disease
EGFR	Epidermal Growth Factor Receptor
ERK	Extracellular Signal-Regulated Kinase
FAK	Focal Adhesion Kinase
GR/NR3C1	Glucocorticoid Receptor
IGF-1R	Insulin-Like Growth Factor 1 Receptor
IL-1β, IL-6, IL-8, IL-10	Interleukins
JAK	Janus Kinase
JNK	c-Jun N-Terminal Kinase
MAPK	Mitogen-Activated Protein Kinase
MGD	Meibomian Gland Dysfunction
MUC5AC	Mucin 5AC
NF-κB	Nuclear Factor Kappa B
NGF	Nerve Growth Factor
OSD	Ocular Surface Disease
OSDI	Ocular Surface Disease Index
ROS	Reactive Oxygen Species
RT	Radiotherapy
STAT	Signal Transducer and Activator of Transcription
TBUT	Tear Break-Up Time
TED	Thyroid Eye Disease
TGF-β	Transforming Growth Factor Beta
TNF-α	Tumor Necrosis Factor Alpha
TSHR	Thyroid-Stimulating Hormone Receptor
VIP	Vasoactive intestinal peptide
ZO-1	Zonula occludens-1
